# Evaluation of Different Body Cooling Strategies for Race Car Drivers

**DOI:** 10.51894/001c.144125

**Published:** 2025-09-30

**Authors:** Aidan R. Davis

**Affiliations:** 1 College of Osteopathic Medicine Michigan State University

## 40

### INTRODUCTION

There is minimal scientific literature on the physiological responses of race car drivers. However, consistent data demonstrates that thermal strain is the most significant stressor experienced by race car drivers during competition. The issue of thermal strain experienced by drivers came to the public’s attention at the 2023 Formula 1 Qatar Grand Prix where several drivers succumbed to heat illness.

### PURPOSE

Hence the purpose of this investigation was to evaluate various cooling methods for drivers.

### METHODS

Fourteen participants with fitness levels equal to elite racing drivers completed six different exercise trials in a hot humid environment to mimic the race car cockpit. Participants wore appropriate FIA-approved racing attire while cycling on an ergometer for 60 minutes at a heart rate of 65-75% maximum. Heart rate, skin temperature, core body temperature, physiological strain index (PSI) and pre/post-exercise nude body weight were measured (to determine sweat loss). The six exercise trials included (1) Control (no cooling), (2) Cool Shirt, (3) Helmet Blower with cold air, (4) Suit Blower with cold air, (5) Helmet Blower with ambient air and (6) Suit Blower with ambient air.

### RESULTS

Significant physiological differences were observed between cooling methods (P<0.001). Heart rate, skin temperature, core temperature, and PSI increased during all trials, but the Suit Blower-cold air and Helmet Blower-cold air proved most effective at maintaining the lowest core and skin temperatures relative to the Control condition. In contrast, the Cool Shirt, Suit Blower-ambient air, and Helmet Blower-ambient air were less effective.

### CONCLUSION

The Suit Blower that introduced cold air into the driver’s suit was the most effective at reducing thermal strain.

